# Secondary Syphilis Presenting as Erythema Multiforme in the Setting of AIDS and Psoriasis

**DOI:** 10.7759/cureus.29110

**Published:** 2022-09-13

**Authors:** Olivia M Cook, Jenna Knafo, Rahill Bhaskar, Mohammad Salhab, Hoang Nguyen

**Affiliations:** 1 Medicine, Nova Southeastern University Dr. Kiran C. Patel College of Osteopathic Medicine, Clearwater, USA; 2 Basic Sciences, Nova Southeastern University Dr. Kiran C. Patel College of Osteopathic Medicine, Clearwater, USA

**Keywords:** acquired immune deficiency syndrome (aids), treponema pallidum, targetoid, bacterial sexually transmitted infections, skin disease/ dermatology, "psoriasis, cutaneous syphilis, human immunedeficiecy virus (hiv) infection

## Abstract

Syphilis is a highly infectious sexually transmitted infection (STI) with a multitude of presentations. The disease is known as "the great imitator" as it often presents as other chronic dermatoses, leading to a difficult and delayed diagnosis. Here, we describe the case of a 17-year-old Vietnamese male from Dong Nai Province who was initially diagnosed with psoriasis. However, upon further investigation, he was found to have concurrent secondary syphilis and psoriasis complicated by an undiagnosed human immunodeficiency virus (HIV) infection which presented clinically as generalized erythema multiforme (EM). The patient demonstrated significant improvement after being treated for syphilis and psoriasis, and he was subsequently referred to an infectious disease specialist for treatment of the underlying HIV infection.

## Introduction

*Treponema pallidum*, the causative organism of syphilis, often presents with rashes that mimic various dermatological diseases, earning its reputation as "the great imitator" [1]. The spirochete is transmitted through direct contact with active lesions during sexual activities or transplacentally from mother to fetus in pregnancy. Primary syphilis infection presents with a painless genital chancre or ulcer at the initial site of infection. Untreated, syphilis progresses to the secondary stage, which presents with a mucocutaneous rash, often mimicking psoriasis, drug eruptions, pityriasis rosea, and other dermatoses, along with lymphadenopathy and nonspecific flu-like symptoms. Following a latent period, tertiary syphilis can occur, leading to neurosyphilis, destruction of the heart and vasculature, and gummata. Gummata, which are characteristic of advanced stages of syphilis, appear in skin, bone, and internal organs as single or multiple coalescing plaques or nodules with central ulceration and scarring [2]. In rare circumstances, immunocompromised patients, such as those with human immunodeficiency virus (HIV) or acquired immunodeficiency syndrome (AIDS), co-infected with syphilis, may present with targetoid lesions resembling erythema multiforme (EM) [3-5]. EM is a self-limiting type IV hypersensitivity reaction, a cell-mediated reaction that occurs in response to contact with allergens. EM is most often triggered by the herpes simplex virus (HSV), Mycoplasma pneumoniae, and certain medications. Clinically, it is characterized by target or iris lesions [6].

Though the incidence of syphilis markedly declined in the 1980s and 1990s, it remained endemic in developing countries such as Vietnam. However, in recent years, the incidence has drastically increased globally, highlighting the importance of recognizing the diverse manifestations of this sexually transmitted infection (STI) [7,8]. Here, we present an HIV-positive patient with secondary syphilis who presented clinically with erythema multiforme and histopathologically with psoriasis, highlighting *T. pallidum* as "the great imitator."

## Case presentation

In late 2020, a 17-year-old Vietnamese male presented to Ho Chi Minh City Hospital of Dermato Venereology in Ho Chi Minh City, Vietnam with a chief complaint of red, scaly skin. Although previously healthy, a thorough history revealed this was his third time seeking medical attention for dermatologic complaints. He first presented to a different physician nine months prior with erythematous lesions on his hands and feet, a fever, and lower extremity pain. He reported being treated with intravenous (IV) fluids and a single dose of intramuscular ketorolac, which resulted in a transient resolution of symptoms. Unfortunately, the patient was unable to recall additional details about his diagnosis and workup. Three months following his initial presentation, he presented to another physician with erythematous plaques and overlying thick, silvery scales distributed along his scalp, trunk, extensor elbows, knees, genitals, ankles, and feet. He was diagnosed with psoriasis and was prescribed an unknown medication. However, he reported noncompliance with the medication and utilized unknown East Asian medicine alternatives instead.

**Figure 1:**
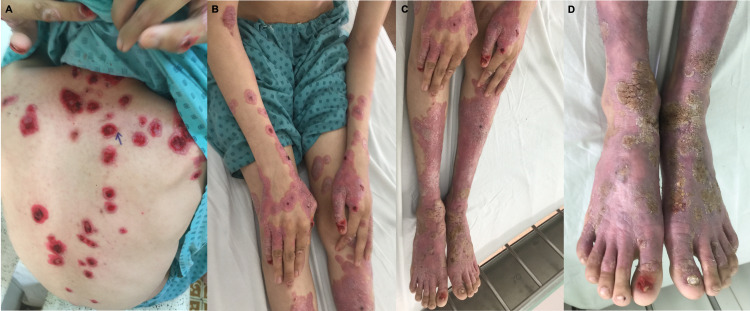
Skin examination revealed numerous well-demarcated, annular, erythematous, and violaceous papules and plaques resembling erythema multiforme on the face, trunk, genitals, and extremities. (A) Truncal lesions appeared targetoid with central crusted erosions. (B, C) Confluent patches with significant scales and (D) brown crusts were noted on the extremities, and nails demonstrated erosion.

Upon presentation to Ho Chi Minh City Hospital of Dermato Venereology, his physical examination demonstrated a non-pruritic, generalized, heterogeneous rash, divergent from his presentations nine and six months prior, respectively. There were numerous well-demarcated, annular, erythematous, and violaceous papules and plaques distributed across the face, trunk, genitals, and extremities. Many lesions located on the trunk and extremities appeared targetoid, some with central crusted erosions, resembling erythema multiforme. Confluent patches with significant scales and brown crusts were noted, especially on the forearms, hands, lower legs, and feet. Several nails appeared eroded as well (Figure 1). He denied having contact with anyone with similar symptoms and denied a history of risky sexual behavior or IV or other illicit drug use. The patient’s vital signs were stable. There was no mucosal involvement or evidence of organ damage, and the physical exam was otherwise unremarkable. A complete blood count (CBC) revealed mild microcytic anemia, and serum chemistry demonstrated mildly decreased high-density lipoprotein (HDL) (Table 1).

**Table 1:** Serum Laboratory Test Results WBC: white blood cell; RBC: red blood cell; MCV: mean corpuscular volume; MCH: mean corpuscular hemoglobin; MCHC: MCH concentration; RDW: RBC distribution width; MPV: mean platelet volume; HDL: high-density lipid; LDL: low-density lipid; ALT: alanine aminotransferase; AST: aspartate aminotransferase; RPR: rapid plasma reagin; TPHA: *T. pallidum* hemagglutination assay; HIV rapid test: 3rd-generation VIKIA HIV 1/2 rapid test HIV antigen/antibody assay: 4th-generation ADVIA centaur HIV combo assay

Test name	Result	Reference range
Complete blood count		
WBC (#)	7.23	4.6–10.2 × 10^9^/L
Neutrophil (%)	70.86	37–80%
Lymphocyte (%)	12.84	10–50%
Monocyte (%)	12.49	0–12%
Eosinophil (%)	3.27	0–7%
Basophil (%)	0.55	0–2.5%
Neutrophil (#)	5.12	1.6–7.0 × 10^3^/mm^3^
Lymphocyte (#)	0.93	1.0–3.0 × 10^3^/mm^3^
Monocyte (#)	0.9	0.2–0.8 × 10^3^/mm^3^
Eosinophil (#)	0.24	0.0–0.7 × 10^3^/mm^3^
Basophil (#)	0.04	0.0–0.2 × 10^3^/mm^3^
RBC (#)	4.41	4.0–6.13 × 10^12^/L
Hemoglobin	10.14	12.0–18.1 g/dL
Hematocrit	32.84	37.0–53.7%
MCV	74.51	80–97 fL
MCH	23.01	27.0–31.2 pg
MCHC	30.88	30–36 g/dL
RDW	11.93	10–15%
Platelet	355.2	142–424 × 10^9^/L
MPV	5.32	5.5–11.0 fL
Serum biochemistry		
Glucose	6.08	3.9–6.4 mmol/L
Creatinine	61.6	44–106 μmol/L
Cholesterol	3.02	0.0–5.7 mmol/L
HDL	0.6	0.90–1.68 mmol/L
LDL	1.82	0.0–4.0 mmol/L
Triglyceride	1.34	<1.7 mmol/L
ALT	20	5–40 U/L
AST	23	5–40 U/L
Serum electrolytes		
Potassium	3.83	3.5–5.3 mmol/L
Sodium	138.37	135–148 mmol/L
Chloride	99.32	95–108 mmol/L
Calcium	1.29	1.17–1.29 mmol/L
Additional tests		
RPR	Reactive 1:512	Non-reactive
TPHA	Positive	Negative
HIV rapid test and antigen/antibody assay	Positive	Negative
CD4+ T-cell (#)	187	500–1500/mm^3^

The patient was treated with amoxicillin/clavulanic acid 2 g/day, acitretin 50 mg/day, vitamin E 4000 IU/day, chlorpheniramine 4 mg/day, zinc 20 mg/day, topical salicylic acid 5%, eosin 2%, and calcipotriol/betamethasone for suspected psoriasis with overlying skin infection pending additional laboratory and pathology evaluation. Skin biopsies from the posterior trunk and lower leg revealed epidermal hyperplasia of the stratum spinosum and stratum corneum with the presence of Munro-Saboureau microabscesses, collections of neutrophils in the epidermal stratum corneum, which are pathognomonic for psoriasis. There was also evidence of chronic inflammatory infiltrate with vascular proliferation in the dermal papillae, suggesting psoriasis with an overlying superinfection. A biopsy from the patient’s right ankle similarly exhibited epidermal hyperplasia of the stratum spinosum and stratum corneum. However, it also demonstrated spongiosis, numerous neutrophils, and extensive plasma cell infiltration, raising suspicion of syphilis. The immunofluorescence assay of the specimen was negative for IgG, IgA, IgM, fibrinogen, and C3. Following biopsy results, *T. pallidum* rapid plasma reagin (RPR), *T. pallidum* hemagglutination assay (TPHA), and a rapid HIV antibody blood test were performed. RPR was reactive at a titer of 1:512, and TPHA was positive +++ (a reaction of ++ or greater indicates a positive result). The positive RPR and TPHA tests, along with the biopsy results, confirmed a diagnosis of syphilis. Additionally, a third-generation VIKIA HIV-1/2 rapid test and a fourth-generation ADVIA Centaur HIV antigen/antibody combo assay were both positive. The CD4+ T-cell count was 187 cells/mm3, suggesting a diagnosis of AIDS. However, the patient denied a past or present history of opportunistic infection.

**Figure 2:**
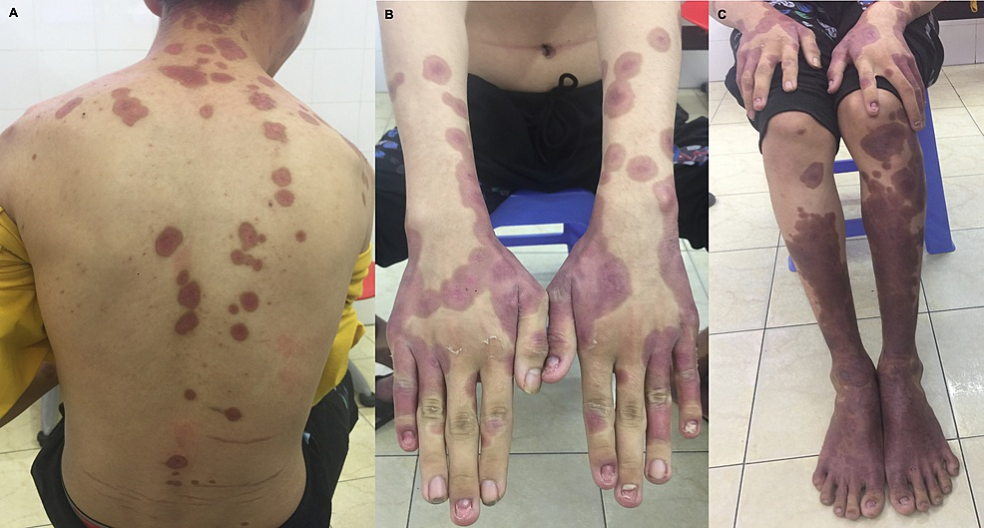
Skin examination one week post-discharge revealed significant healing of lesions with post-inflammatory hyperpigmentation on the face (A) trunk, and (B, C) upper and lower extremities.

One dose of benzathine penicillin G 2.4 million units intramuscularly (IM) was given to treat the syphilis infection, and acitretin, salicylic acid, calcipotriol/betamethasone, and vitamin E were continued to treat psoriasis. Chlorpheniramine was also continued for symptomatic treatment. All other medications were discontinued. Following treatment, the lesions revealed significant improvement, and the patient was discharged on day 17 of hospitalization. At a one-week follow-up, only post-inflammatory hyperpigmented patches remained, but the crusts and erosions had resolved (Figure 2). The patient was advised to continue acitretin and calcipotriol/betamethasone for psoriasis and to follow up with an infectious disease specialist for HIV treatment and further diagnostic testing for opportunistic infections. Unfortunately, the patient was lost to follow-up, and his current state of health is unknown.

## Discussion

Here, we describe a case of serology-confirmed secondary syphilis and pathology-confirmed psoriasis in an HIV-positive patient presenting clinically with erythema multiforme-like lesions. This patient’s unique presentation allowed an opportunity to observe the dermatologic manifestations of numerous maladies at varying degrees in their course.

Secondary syphilis presents with a wide variety of clinical manifestations, typically including cutaneous lesions and nonspecific constitutional symptoms within three months of exposure [9]. Interestingly, severe, persistent, and atypical courses of syphilis frequently occur in patients with concurrent HIV/AIDS [10,11]. This is especially true in patients with very low CD4+ T-cell counts [12]. The immune response to an initial syphilis infection is chiefly mediated by CD4+ T-cells, which favor the elimination of the organism via a well-developed chancre. HIV-induced CD4+ T-cell destruction hinders this response and may lead to asymptomatic primary infection. A weakened humoral response and a relatively heightened CD8+ T-cell response result in prolonged infection and progression to advanced syphilitic stages, such as what was seen in this case [13].

To date, there are few cases of EM-like eruptions in patients with syphilis and HIV in the literature [3-5]. Due to the rarity and the multitude of dermatological presentations of secondary syphilis, EM is not usually the first consideration among clinicians, as demonstrated in our case. The mechanism of EM-like eruptions in patients with syphilis is not well understood, but it has been hypothesized that the rash may arise due to an allergic reaction [14]. The lesions of EM are usually triggered by HSV or M. pneumoniae. Unfortunately, HSV and M. pneumoniae tests were not obtained in our patient. However, at the time of presentation, the patient did not demonstrate respiratory symptoms, lymphadenopathy, pharyngitis, fever, or other systemic features suggestive of another infectious etiology. Cytomegalovirus (CMV) may be another cause of EM. Although CMV testing was not performed either, the patient’s CD4+ T-cell count of 187 cells/mm3 made CMV infection unlikely. Despite the absence of the typical triggers, our patient presented with a classic appearance of EM-like targetoid lesions with distinct borders and three concentric zones: an erythematous, dusky, and sometimes crusted or blistered center; a surrounding pale, edematous ring; and a pale erythematous periphery [15]. Furthermore, this patient was not evaluated for cutaneous fungal infection via potassium hydroxide (KOH) preparation or fungal staining, a condition that likely should have been considered in a patient with concurrent syphilis and HIV. Additionally, this patient should have also been tested for concurrent Gonorrhea and Chlamydia sexually transmitted infections. However, our patient’s rapid clinical improvement following penicillin treatment suggests that *T. pallidum* was the causative pathogen of the EM-like eruption.

Though largely variable, the histological pattern of syphilitic lesions often demonstrates a nonspecific psoriasiform or lichenoid inflammatory reaction with the presence of perivascular plasma cell infiltrate and endothelial proliferation [16]. EM typically presents with basal layer vacuolization with keratinocyte necrosis, which we did not observe in our patient [17]. Rather, we initially observed histological findings pathognomonic for psoriasis; however, additional biopsy of a lesion from the patient’s ankle demonstrated a pattern suggestive of concurrent syphilis. Although spirochetes were not found on skin biopsy, the clinical presentation and serological testing substantiated a diagnosis of secondary syphilis.

## Conclusions

The complex interactions between autoimmune and infectious diseases in the setting of immunodeficiency present challenges even to experienced clinicians. Furthermore, the variable presentation of concurrent diseases often leads to a delay in diagnosis and, ultimately, treatment. With any skin manifestation, a detailed history may clue a clinician into a disease course that may be atypical. Additional testing, such as histopathology and tests specific to infectious etiology, should be employed in patients with persistent and complex symptoms despite treatment. Given this patient’s denial of risky sexual behavior or illicit drug use, STIs and HIV were not initially considered in the differential diagnosis. This case was further complicated by unknown treatments from prior clinicians and a nine-month delay in treatment due to the patient’s decision to pursue East Asian Medicine alternatives. There is no clear way to evaluate the effects any of these treatments may have had on the patient.

Given the increasing incidence of syphilis worldwide and its tendency to mimic other illnesses, dermatologists must become familiar with its numerous cutaneous manifestations, including those that deviate from typical patterns, such as erythema multiforme-like lesions. Furthermore, syphilis should be considered as a potential precipitating or contributing factor in patients presenting with generalized EM, especially in those with HIV/AIDS.
